# A SNP Based Linkage Map of the Arctic Charr (*Salvelinus alpinus*) Genome Provides Insights into the Diploidization Process After Whole Genome Duplication

**DOI:** 10.1534/g3.116.038026

**Published:** 2016-12-16

**Authors:** Cameron M. Nugent, Anne A. Easton, Joseph D. Norman, Moira M. Ferguson, Roy G. Danzmann

**Affiliations:** Department of Integrative Biology, University of Guelph, Ontario N1G 2W1, Canada

**Keywords:** diploidization, duplicated genes, epigenetic modification, linkage map, salmonid fishes, transmission genetics, transposition

## Abstract

Diploidization, which follows whole genome duplication events, does not occur evenly across the genome. In salmonid fishes, certain pairs of homeologous chromosomes preserve tetraploid loci in higher frequencies toward the telomeres due to residual tetrasomic inheritance. Research suggests this occurs only in homeologous pairs where one chromosome arm has undergone a fusion event. We present a linkage map for Arctic charr (*Salvelinus alpinus*), a salmonid species with relatively fewer chromosome fusions. Genotype by sequencing identified 19,418 SNPs, and a linkage map consisting of 4508 markers was constructed from a subset of high quality SNPs and microsatellite markers that were used to anchor the new map to previous versions. Both male- and female-specific linkage maps contained the expected number of 39 linkage groups. The chromosome type associated with each linkage group was determined, and 10 stable metacentric chromosomes were identified, along with a chromosome polymorphism involving the sex chromosome AC04. Two instances of a weak form of pseudolinkage were detected in the telomeric regions of homeologous chromosome arms in both female and male linkage maps. Chromosome arm homologies within the Atlantic salmon (*Salmo salar*) and rainbow trout (*Oncorhynchus mykiss*) genomes were determined. Paralogous sequence variants (PSVs) were identified, and their comparative BLASTn hit locations showed that duplicate markers exist in higher numbers on seven pairs of homeologous arms, previously identified as preserving tetrasomy in salmonid species. Homeologous arm pairs where neither arm has been part of a fusion event in Arctic charr had fewer PSVs, suggesting faster diploidization rates in these regions.

Whole genome duplications (WGDs) are rare evolutionary events that drastically alter genomic architecture by producing duplicate copies of every chromosome. The doubling of all loci can be a major driving force of evolution, as there is the potential to produce loci with novel functions through the accumulation of mutations that were formerly selected against (Ohno 1970). WGDs provide a surfeit of genetic information that can be associated with adaptive innovation and evolutionary change (Taylor and Raes 2004; Moghadam *et al.* 2005; Nakatani *et al.* 2007; Berthelot *et al.* 2014). WGDs have been important events in the evolutionary history of vertebrate animals (Smith *et al.* 2013). Two WGDs occurred in the common ancestor of all vertebrates, and these (referred to as the 1R and 2R WGDs) yielded a karyotype of between 40 and 52 chromosomes from the protovertebrate karyotype of 10–13 chromosomes (Nakatani *et al.* 2007). A third (3R) WGD occurred ∼400 million yr ago (MYA) in the ancestor of teleost fish, while a fourth salmonid specific WGD (Ss4R) took place ∼96 MYA in the ancestor of salmonid fishes (Allendorf and Thorgaard 1984; Berthelot *et al.* 2014; Macqueen and Johnston 2014; Lien *et al.* 2016). Subsequent chromosome fusions and fissions caused changes in chromosome numbers and genomic architecture in specific vertebrate lineages (Kasahara *et al.* 2007; Nakatani *et al.* 2007).

Following WGD, the genome undergoes the process of diploidization, where it reverts from a tetraploid (4*n*) to a diploid (2*n*) state. Specifically, chromosome pairs that shared a common genetic ancestor prior to WGD (termed homeologs), diverge from one another due to genomic rearrangements, gene deletion, pseudogenization, and mutation (Comai 2005; Bergthorsson *et al.* 2007). Salmonids are in the midst of the diploidization process, in that some regions of the genome have diverged into two pairs of diploid loci, while in other regions, residual tetrasomy occurs as the result of multivalent formation and recombination among homeologues during meiosis (Berthelot *et al.* 2014; Allendorf *et al.* 2015; May and Delany 2015; Lien *et al.* 2016). For example, 52% of genes in the rainbow trout (*Oncorhynchus mykiss*) genome have diploidized since the Ss4R WGD, while the other 48% of genes have retained both copies, and have yet to revert to a diploid state (Berthelot *et al.* 2014). Similarly, work on the Atlantic salmon (*Salmo salar*) genome has found that 55% of genes have been retained as two functional copies since the Ss4R WGD (Lien *et al.* 2016). Chromosomal architecture appears to play a role in determining which genomic regions undergo homeologous recombination during meiosis in these fishes (Brieuc *et al.* 2014; Lien *et al.* 2011; Kodama *et al.* 2014; Waples *et al.* 2015). All recombining pairs of homeologs appear to include one large chromosome, either a metacentric produced through the Robertsonian fusion of two chromosome arms, or a fused acrocentric resulting from the tandem fusion of two smaller acrocentric chromosomes. This suggests that the large size of these chromosomes may provide the stability necessary for homeologous recombination (Brieuc *et al.* 2014; Kodama *et al.* 2014; Lien *et al.* 2016). Furthermore, the telomeric regions of homeologs show the slowest rates of diploidization within several salmonid species, as illustrated by a relatively high number of paralogous loci (Brieuc *et al.* 2014; Waples *et al.* 2015; Lien *et al.* 2016).

The development of detailed linkage maps has shown that lineage specific changes in chromosomal architecture have taken place in salmonids following WGD. Maps are available for species of *Salmo* and *Oncorhynchus* (Rexroad *et al.* 2008; Gonen *et al.* 2014; Limborg *et al.* 2015; McKinney *et al.* 2015; Waples *et al.* 2015; Tsai *et al.* 2016). In the karyotypes of certain species such as Atlantic salmon, a large number of chromosomes are the product of fusions (*e.g.*, 42/58) (Phillips and Ráb 2001). Variation in genomic architecture may cause the rate of diploidization to vary across the genomic landscape of the different taxa, and which regions of the genome undergo residual tetrasomy (Lien *et al.* 2011; Brieuc *et al.* 2014; Kodama *et al.* 2014; Lien *et al.* 2016). Similar studies of taxa with more basal karyotypes, such as the *Salvelinus* species, would improve our understanding of the role that genomic rearrangements have in the diploidization process. For instance, karyotype data suggests that only 20 of the 78 chromosomes (2*n* = 78) in Arctic charr (*Salvelinus alpinus*) are metacentric, suggesting that fewer Robertsonian fusion events have occurred in their evolutionary history (Hartley 1989; Phillips *et al.* 2002). The study of genomic evolution in Arctic charr is currently limited by the low resolution of available genetic linkage maps, and the paucity of known molecular markers (Woram *et al.* 2004; Norman *et al.* 2011; Timusk *et al.* 2011). Expanding the genomic resources of Arctic charr through the addition of several thousand SNPs will make it possible to track the genomic rearrangements that have shaped the modern Arctic charr karyotype.

The initial aims of this study were to (1) increase the number of known genetic markers in the Arctic charr genome using genotype by sequencing (GBS); (2) create a second generation genetic linkage map of the Arctic charr genome using newly identified SNP markers, and integrate the revised map to previous versions primarily based on microsatellite loci; and (3) identify the chromosome type associated with each linkage group, and use this information to test for the existence of acrocentric-acrocentric homeologous pairs in the genome. These are of interest because a lack of homeologous recombination could cause diploidization to occur faster in homeologous pairs of this type. Using the data produced to meet the above goals, we also aimed to (4) compare genomic data from Arctic charr to the genomes of rainbow trout and Atlantic salmon to identify chromosome arm homologies across these three species, allowing the characterization of conserved genomic rearrangements and fusion/fission events unique to the Arctic charr lineage; and, also, (5) identify putative duplicate loci, and assess their distribution across the Arctic charr genome. As we analyzed sequence data, it became apparent that there were signatures of significant transposon activity within the Arctic charr genome. We therefore used this opportunity to characterize transposable element (TE) activity in the Arctic charr genome, and see how TE distribution is affected by residual tetrasomy, chromosome architecture, and the uneven distribution of duplicate loci throughout the genome.

Transposon activity is associated with important evolutionary transitions, adaptation to novel environments, and extensive changes in genome evolution (de Boer *et al.* 2007; Schrader *et al.* 2014; Staton and Burke 2015). In fact, the estimated time of radiation of *Salmoninae* into *Salmo*, *Oncorhynchus*, and *Salvelinus* (14–23 MYA) (Macqueen and Johnston 2014) coincides with a known spike in TE activity (de Boer *et al.* 2007). TEs cause sequence deletion or duplication due to unequal homologous recombination and segmental duplication, and they facilitate genomic rearrangements (Kazazian 2004). Genomic regions with an accumulation of TEs appear to evolve faster than the rest of the genome (Schrader *et al.* 2014). Therefore, TE activity might be influenced by the rate of residual tetrasomy in particular chromosomal regions, or vice versa. Reduced presence of TEs has been observed in the duplicated regions of Atlantic salmon, chum salmon (*O. keta*), and chinook salmon (*O. tshawytscha*) that lag behind in the diploidization process (Larson *et al.* 2015; McKinney *et al.* 2015; Waples *et al.* 2015; Lien *et al.* 2016). Therefore, our final goal was to characterize TE activity in the Arctic charr genome, and determine if there is a relationship between residual tetrasomy and TE activity in the telomeric regions of homeologs that undergo homeologous recombination.

## Materials and Methods

### Source mapping panel

The analysis utilized 85 full-siblings, and their parents, from a single family of Fraser strain Arctic charr obtained from the Coastal Zones Research Institute (CZRI), Shippagan, NB Canada. The Fraser strain originated from collections of fish from the Fraser River, Labrador, Canada, in the 1980s. The family was produced on November 6, 2012, and reared communally until March 11, 2014, at which time each fish was PIT tagged, weighed (to the nearest gram), measured (fork length), and samples of adipose fin were removed for DNA analysis. DNA was extracted using a commercial kit (Qiagen DNeasy Blood & Tissue), as per manufacturer’s instructions, and treated with RNase A to remove any RNA. The samples were then quantified using a Qubit Fluorometer to ensure that all DNA concentrations exceeded 50 ng/µl.

### Sequencing analysis

The DNA samples were submitted for GBS (Elshire *et al.* 2011) at the Cornell Institute of Biotechnology. DNA from each progeny was added to a single well of a 96-well plate, while the parents were analyzed in triplicate, to increase sequencing depth and provide the information necessary for linkage mapping based on the inheritance of SNP alleles. The four grandparents of the family were also added to a single well each. Samples were digested with the restriction enzyme *Eco*T22I, and unique barcode sequence adapters (4–8 bp in length) were ligated to each of the DNA samples (three for each parent) such that the DNA sequence data could be assigned to a specific individual or parental subsample. After the barcodes were added, sequencing primers, and the samples from all 95 wells containing DNA samples (and a single blank control well) were pooled. Paired end sequencing primers with oligonucleotides that allow binding to the sequencing flowcell were then added to the pooled samples. Polymerase chain reaction (PCR) was then used to amplify the DNA fragment pool, and the resulting DNA products were analyzed for fragment size. The DNA samples were then sequenced on an Illumina Hisequation 2000 high-throughput sequencing instrument, and replicated across two flowcells. The sequencing process produced 100 bp single-end reads. GBS sequence reads are available in the NCBI sequence read archive (www.ncbi.nlm.nih.gov/sra) under the BioProject accession number #SRP026259 and BioSample accession numbers #SAMN06165956 and #SAMN06165957.

### SNP identification from raw sequence data

Raw sequence reads were analyzed using the UNEAK pipeline, part of the Tassel 3.0 software package produced by the maize genetics laboratory at Cornell University (Glaubitz *et al.* 2014). The UNEAK pipeline allows for the identification of SNPs in species where a reference genome is not available. The 5′ sequence barcodes were used to define individual-specific reads, but were trimmed prior to sequence analysis. Alignment of all the sequence data was conducted to produce a master “tag” list for the dataset. Tags are unique sequences of up to 64 bp in length observed across multiple reads. Further alignment of the master tag list identified tag pairs with a single base pair mismatch, and these were considered SNPs (with a sequencing error tolerance rate parameter of 0.03) (for more information, see Glaubitz *et al.* 2014). Note the program only considered tag pairs with a 1 bp mismatch to be SNPs, so any 64 bp read with 2+ SNPs would be excluded. The number of times each tag is observed in the sequencing data from each individual is used to determine the individual’s genotype for a particular SNP.

### SNP filtering

The inventory list of SNP genotypes identified within the UNEAK pipeline was analyzed manually to remove markers where: (1) data were available for <75/85 progeny; (2) the genotype for one or both of the parents was missing. Markers where SNP inheritance displayed significant segregation distortion (*i.e.*, SNPs with *G* values >6.693, *P* < 0.01) were identified and removed using LINKMFEX (Danzmann 2016).

### Microsatellite genotyping

To anchor the newly identified SNP markers to previous Arctic charr linkage maps, genotypes at 102 microsatellite loci (Supplemental Material, File S1) from known locations across the Arctic charr genome were determined for all progeny and parents using established genotyping methods (Moghadam *et al.* 2007; Timusk *et al.* 2011; Norman *et al.* 2014).

### Linkage mapping

The high quality SNPs selected for mapping, and the microsatellite marker anchors, were assessed for genetic linkage using LINKMFEX (Danzmann 2016). The SNPs were split into three categories: heterozygous male, heterozygous female, and double heterozygote (DH) SNPs where both parents were heterozygotes. The SNPs where only a single parent was heterozygous had high information content, given that all progeny genotypes are informative for linkage mapping. Double heterozygous SNP markers are informative in only about half of the progeny, given that linkage phases cannot be assigned in heterozygous progeny. Markers that are heterozygous in only one parent are problematic in that SNP mapping locations cannot be compared between parents. Data from additional mapping panels will be required to compare map orders between the sexes for these markers. Double heterozygous SNP markers were assigned to specific linkage groups, but were not added to specific map locations.

Linkage groups were identified using a logarithm of odds (LOD) threshold of 10 for linkage group assessment. We first created male-parent- and female-parent-specific linkage maps using the SNPs that were heterozygous in only one parent using LINKMFEX. In order to integrate the male and female linkage maps with one another, SNPs where both parents were heterozygous were added to each of the two datasets, and analyzed in LINKMFEX for genetic linkage with a LOD=10 threshold. It was then possible to identify overlapping sets of male- and female-specific linkage groups.

To determine the location of unlinked markers, repeated analysis was performed using descending LOD scores (LOD = 6 down to LOD = 3) using LINKMFEX, and LOD = 3 additions were accepted if they created joinings of two or more linkage groups that are known to be homeologous to one another. For some markers, a pseudolinkage (see below) of known homeologous chromosome arms was detected within the range of LOD = 3.0 to LOD = 5.0, and were therefore accepted. Linkage groups were named according to historical designations based on previous microsatellite marker assignments (Woram *et al.* 2004; Norman *et al.* 2011; Timusk *et al.* 2011). For each linkage group, marker order was determined in OneMap using the record algorithm (Margarido *et al.* 2007). The record algorithm was selected for ordering because it consistently gave the shortest map distances [*i.e.*, smallest number of adjacent double cross-over (DCO) alignment points] out of the three possible OneMap ordering functions (ug, rcd, and record). This option also gave shorter map lengths than those produced by LINKMFEX. Male and female marker orderings were determined separately for each linkage group. As mentioned above, markers heterozygous in both parents were not ordered. OneMap marker ordering was further refined using the LINKMFEX program Adjacent-DCO-Count_Ripple-Check. This program was used to identify and reorder marker placements causing adjacent DCOs in the ordering. Adjacent double cross-overs are biologically unlikely in salmonids, given the high levels of chromatid interference detected during meiosis (Sakamoto *et al.* 2000; Danzmann and Gharbi 2001; Allendorf *et al.* 2015) in these species. The revised marker ordering minimized the number of adjacent DCOs in the dataset. Final map distances were calculated using the MAPDIS-V program in LINKMFEX, and selecting the option to ignore adjacent DCO events. We chose this option, as we considered that remaining adjacent DCOs may be due to errors in genotyping calls.

### sdY marker

The progeny were genotyped for the sexually dimorphic gene (sdY) located on the Y-chromosome, using the PCR and agarose gel visualization methods described in Yano *et al.* (2012) with two modifications. First, we substituted insulin-like growth factor binding protein 5 (IGFBP5) as a positive control, using primers we developed: DQ206713-F3 (CCACCAGCTAATTACTGCAA) and DQ206713-R3 (GTAGAATTTGGCTGGCCCTA). Second, the following PCR temperature cycling conditions was used: denaturation for 5 min at 95°, followed by five cycles of 95° for 1 min, 58° for 30 sec, and 72° for 30 sec, then 30 cycles of 95° for 30 sec, 58° for 30 sec, and 72° for 30 sec, followed by a final 10 min at 72°. The sdY marker was validated based on conformity of the sdY genotypes to phenotypic assessment of the individual’s sex in this population.

### Comparison with rainbow trout and Atlantic salmon genomes

SNP sequences were compared to the rainbow trout draft genome, and the Atlantic salmon genome (Berthelot *et al.* 2014; Lien *et al.* 2016), using the following BLASTn parameter settings (-word_size 11 -gapopen 5 -evalue 0.00001 -gapextend 2 -reward 2 -penalty -3) (Altschul *et al.* 1990). Blast hits were filtered, and the hits with the lowest *e*-value were used for subsequent homology identification. In the case of equivalent *e*-values, all Blast hits were retained, and considered equal “top hits” for the given SNP.

### Moveable genetic elements and homeologies

Following Blast comparison of the Arctic charr markers to the rainbow trout and Atlantic salmon genomes, SNPs that aligned to adjacent regions of a single rainbow trout, and Atlantic salmon chromosome arm, allowed for the identification of arm homologies across the species. Within the SNP clusters that displayed consistent homology to a given Atlantic salmon, or rainbow trout, chromosome arm, individual markers sometimes showed homologies to disparate regions of the genome. Two hypotheses about the cause of these disparate SNPs were tested: (1) the SNPs lie within moveable, and/or highly repetitive DNA sequences; (2) the SNPs may be aligning to a homeologous chromosome arm with highly similar sequences. To test for the existence of repetitive sequences and moveable DNA elements, BLASTn (same parameters as above) was used to compare the linkage map SNPs to Repbase Update’s database of known vertebrate moveable and repetitive DNA elements (Jurka *et al.* 2005). The distribution of TE Blast hits in the linkage map was used to assess TE activity in the Arctic charr genome. TE activity in each linkage group was determined by assessing the proportion of markers in each linkage group that displayed significant Blast alignments to TEs.

### Genomic architecture and residual tetrasomy

**Figure 1:**
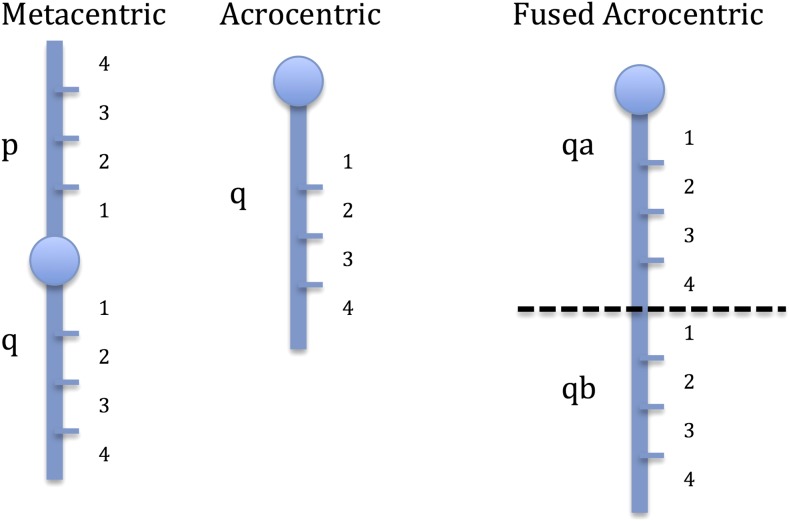
Visual representation of how Atlantic salmon chromosome arms were divided into quarters to assess Arctic charr PSV and map marker BLASTn hit distributions. Circles represent centromeres.

Paralogous sequence variants (PSVs) were identified to assess the distribution of duplicate loci through the Arctic charr genome. To do this, fixed heterozygote SNPs were used as PSV markers. These SNPs were heterozygous in both parents, and 100% of the progeny. The lack of homozygous progeny suggests that these are duplicate, monomorphic, loci with a single base-pair difference, causing them to appear heterozygous in all individuals. Since these PSVs lack recombinant progeny, their location relative to the linkage map SNPs cannot be determined. However, their linkage group affinities can be inferred based upon comparative homologies. We tentatively assigned these PSVs to Arctic charr linkage groups by aligning the PSVs and linkage map SNPs to the Atlantic salmon genome using BLASTn, and comparing their top hit locations. Two characteristics were assessed: (1) the distribution of PSVs between, and within, chromosomes of different genomic architectures, including Acrocentric Homeolog Pairs (AHPs), and High Residual Tetrasomy Arms (HRTAs); (2) the distribution of PSVs along chromosome arms relative to the centromere or telomere (see Figure 1). AHPs were defined as homeologous pairs of Arctic charr chromosome arms where neither arm in the pair is fused with another chromosome arm. HRTAs are defined as pairs of homeologous chromosomes that have higher levels of duplicate loci than the rest of the genome in multiple salmonid species (Danzmann *et al.* 2008; Lien *et al.* 2011, 2016; Brieuc *et al.* 2014), and likely form multivalents during meiosis due to crossing-over between their homeologous arms (Sakamoto *et al.* 2000)

The distribution of duplicate loci in the genome was assessed using the Blast TopHit dataset, which consisted of markers in the Arctic charr linkage map and PSVs. The linkage map markers represent diploid loci, and the PSVs represent tetraploid loci. These markers were assigned to Atlantic salmon chromosome arm based on their top BLASTn hit locations (determined by lowest observed *e*-value). For markers with BLASTn hits of equal *e*-values to a single chromosome arm, only a single hit per chromosome arm was retained in the dataset. In the case of markers (both PSVs and linkage map markers) with equal BLASTn hit locations on two Atlantic salmon chromosome arms, the top Blast hit to each chromosome arm was retained in the dataset. Markers with equal top Blast hits to three or more chromosome arms, and markers with no Blast hits in the Atlantic salmon genome, were removed from the dataset.

To further assess whether any of the apparent single copy SNP markers may be duplicate copies of one another, we performed a BLASTn analysis of all SNPs against all SNPs (non-PSVs) in the database (see parameter settings above). Duplicate pairs exceeding 95% identity, and a 95% overlap in length, and occurring on separate chromosome arms, were considered potential homeologs of one another. Duplicates mapping to the same linkage groups were considered regional marker duplicates, unless they mapped adjacent to one another, indicating some type of tandem duplication.

We tested if duplicate loci are preserved in a higher frequency on HRTAs. Using Atlantic salmon as a reference, the HRTA homeolog pairs are represented by: Ssa02p/Ssa05q, Ssa11qa/Ssa26, Ssa16qa/Ssa17qa, Ssa03q/Ssa06p, Ssa12qa/Ssa02q, Ssa07q/Ssa17qb, and Ssa04p/Ssa08q. Using a contingency chi-square test, the number of linkage map and PSV top BLASTn hits on the HRTAs was compared to the number of hits on all other chromosome arms.

An additional contingency chi-square test was performed to test if Arctic charr AHPs preserve duplicate loci in the same manner as the rest of the genome. Putative AHPs were identified based on homologies in the Arctic charr genome, and their respective Atlantic salmon (Lien *et al.* 2016), and rainbow trout (Danzmann *et al.* 2008; Berthelot *et al.* 2014), homeologies. Based on this information, the following AHPs were identified in Arctic charr: AC02/AC36, AC05/AC29, AC19/AC32, and AC30/AC31. A contingency chi-square test compared the number of linkage map and PSV top BLASTn hits on acrocentric homeolog chromosome arms to the number of hits on all other chromosome arms.

The base pair hit location (s.start) for each marker in the Blast TopHit dataset was used to assess the PSV and linkage map markers’ distributions along Atlantic salmon chromosome arms. Each Atlantic salmon chromosome arm was divided equally into four quarters based on the known base-pair start and end locations (Lien *et al.* 2016). Quarter 1 was closest to the centromere, and quarter 4 was telomeric (Figure 1). All marker hits were then assigned to a chromosome arm quarter, and data from all the chromosome arms were merged. A chi-square test (goodness-of-fit) was performed to see if the frequency of linkage map SNPs and PSVs varied across different chromosome arm quarters. In addition to the above tests, BLASTn was used to compare the PSVs to Repbase update’s list of vertebrate TEs. The number of TE hits in PSVs was then compared to the number of TE hits in the linkage map SNPs using a contingency chi-square test.

We tested if PSVs in Arctic charr aligned more toward the telomeres of Atlantic salmon chromosome arms compared to linkage map SNPs. Tetrasomy is more readily preserved near the telomeres of chromosomes and most cross-overs in multivalent chromosome formations occur toward the telomeres (Sakamoto *et al.* 2000). Therefore, residual tetrasomic inheritance would likely persist in these regions for a longer period of time following the Ss4R WGD, and highly similar duplicate loci are expected to be found in high frequency closer to the telomeres of chromosome arms (Brieuc *et al.* 2014; Allendorf *et al.* 2015).

### Data availability

Details on data used in this study can be found in Materials and Methods under the Sequencing Analysis section, and in the supplementary files.

## Results and Discussion

### SNP identification and linkage mapping

GBS of the Arctic charr family produced 4 × 10^8^ sequence reads (roughly 4.7 million reads per progeny). Using the UNEAK pipeline (Tassel 3.0), 19,418 SNPs were then identified. Following the SNP filtering process, and the addition of microsatellite markers, the mapping dataset consisted of 4536 markers (see File S1 for the DNA sequence corresponding to each SNP marker).

**Figure 2:**
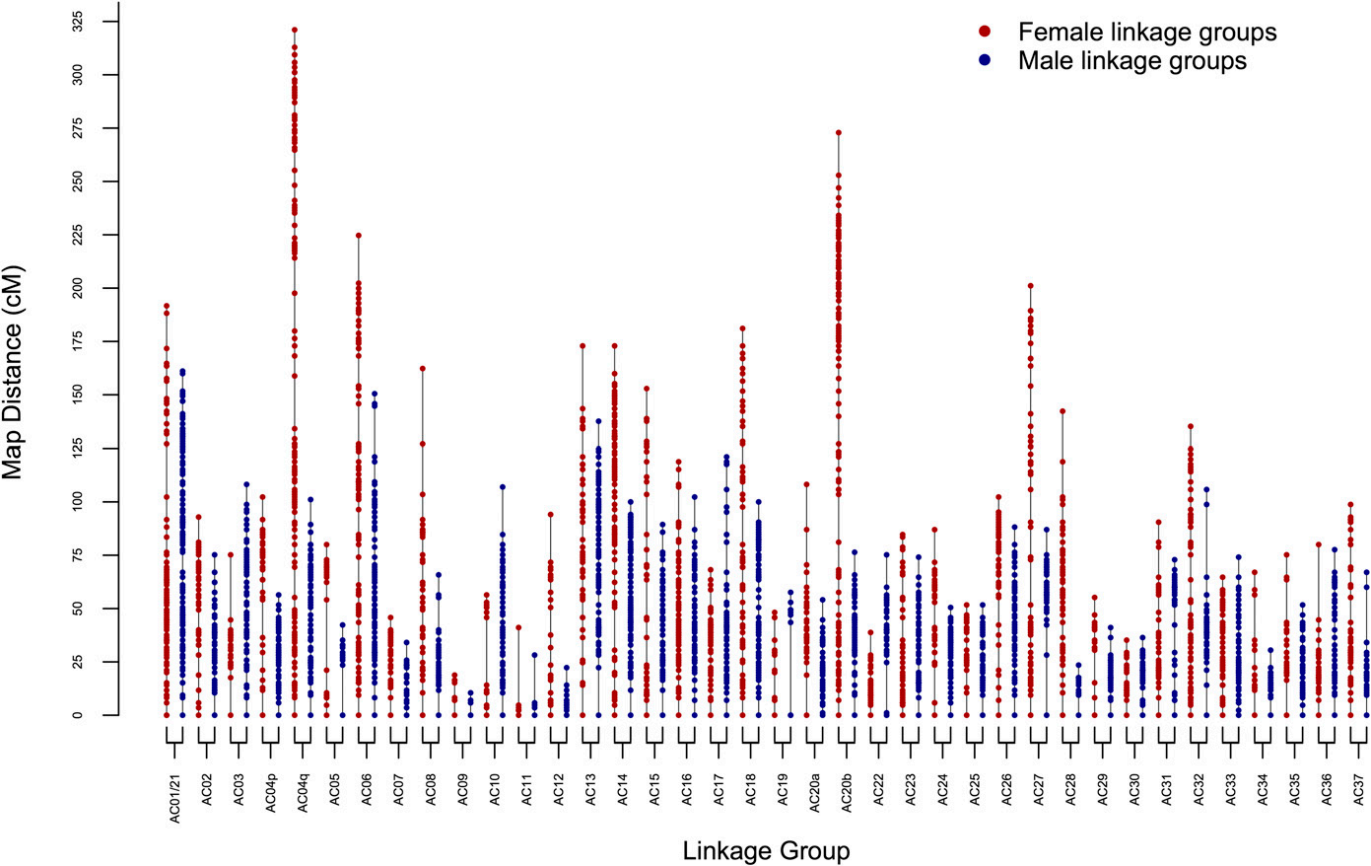
Visual representation of Arctic charr linkage groups. Female linkage groups are shown in red, and male linkage groups are shown in blue. Each point along the length of the line represents a single marker, or zero recombination cluster of several markers.

The linkage mapping process produced 39 linkage groups containing 4508 markers (4405 SNPs, 1 sdY, and 102 SSR), while 28 markers (24 SNP and 4 SSR) remained unlinked. Separate male and female maps were produced (File S2). A total of 1538 markers was ordered in the male map, spanning a distance of 2808.5 cM, while 1709 markers were ordered in the female map, covering 4302.7 cM (Figure 2 and Table 1). Markers that were heterozygous in both parents were not ordered, as mentioned previously, but 1283 of these were assigned to linkage groups (linkage group assignments are found in File S2).

**Table 1:** List of Arctic charr linkage groups; the number of markers in each linkage group and the map distance covered (centiMorgans) by each male and female linkage group

Linkage Group	Male Marker Number	Male Distance (cM)	Female Marker Number	Female Distance (cM)	Unordered Markers	Chromosome Type
AC01/21	116	161.1	94	268.1	92	AC01 is M/AC21 is A
AC02	52	75.2	39	92.9	47	A
AC03	55	108.2	24	75.2	34	M
AC04p	39	56.4	41	102.3	22	A/split M
AC04q	55	101.1	132	321.1	43	Fused A/split M
AC05	12	42.3	22	80	85	A
AC06	74	150.5	102	224.7	33	M
AC07	23	34.1	24	45.8	29	A
AC08	38	65.8	46	162.3	24	M
AC09	4	10.5	9	18.8	76	A
AC10	50	107	15	56.4	13	A
AC11	5	28.2	8	41.1	10	A
AC12	12	22.3	24	94.1	10	A
AC13	73	137.6	55	172.9	18	M
AC14	74	100	87	172.9	47	M
AC15	49	89.4	46	152.9	76	M
AC16	51	102.3	65	118.8	27	A
AC17	46	121.1	40	68.2	19	Fused A
AC18	68	100	62	181.1	34	M
AC19	9	57.6	17	48.2	78	A
AC20a	37	54.1	37	108.2	7	A
AC20b	47	76.4	128	272.9	38	M
AC22	31	75.2	31	38.8	20	A
AC23	45	74.1	38	84.7	25	A
AC24	35	50.5	33	87	6	A
AC25	31	51.7	24	51.7	18	A
AC26	50	88.2	43	102.3	57	A
AC27	34	87	61	201.1	34	M
AC28	16	23.5	48	142.3	9	A
AC29	28	41.1	17	55.2	37	A
AC30	27	36.4	26	35.2	3	A
AC31	35	72.9	40	90.5	33	A
AC32	37	105.8	67	135.2	30	A
AC33	48	74.1	44	64.7	62	A
AC34	22	30.5	18	81.1	8	A
AC35	36	51.7	24	75.2	29	A
AC36	47	77.6	32	80	30	A
AC37	27	67	46	98.8	20	A
Totals	1538	2808.5	1709	4302.7	1283*^a^*	Metacentric: 10*^b^*
						Acrocentric: 27–29
						Split meta: 1*^c^*

The chromosome type [metacentric (M) or acrocentric (A)] is also shown for each linkage group.

a Note that there are 4508 markers in the linkage map, but the total marker numbers here sum to 4530. This is because a small number of markers were successfully ordered in both the male and female maps, and are therefore counted twice in this row.

b 10 metacentric assumes AC20b is metacentric in structure, and in karyotypes where AC04p/q are joined, 11 metacentrics would be observed.

c 27 acrocentrics would be observed if AC20b is metacentric, and AC04 was metacentric in the karyotype, while 29 acrocentrics would be present in the configuration where AC04p and AC04q form separate arms. “Fused A” designations indicate acrocentric arms that appear to be composed to two ancestral teleost chromosome arms.

### Microsatellite anchors

Of the 102 microsatellite markers genotyped, 98 were successfully added to the linkage map (four remained unlinked). Five markers were duplicated and mapped to two linkage groups. Of 39 linkage groups, 36 contained one or more microsatellite markers, allowing the new SNP-based linkage groups to be aligned with the microsatellite-based linkage maps (Woram *et al.* 2004; Timusk *et al.* 2011; Norman *et al.* 2012). The identity of three linkage groups without microsatellite markers (AC25, AC30, and AC31) was determined based on BLASTn search results, and previously identified arm homologies among the salmonid species compared.

### Chromosome type and salmonid arm homologies

A comparison of the Arctic charr linkage map to the Atlantic salmon (Lien *et al.* 2016) and rainbow trout genomes (Berthelot *et al.* 2014) based on the BLASTn analysis is presented in Table 2. The top Blast hit data for each marker are available in File S2 and File S3. Nine linkage groups (AC01, -03, -06, -08, -13, -14, -15, -18, and -27) were identified as metacentric chromosomes based on homologies to two salmonid chromosome arms. Two potential split metacentric groupings were identified (AC04 and AC20). One set involves the sex-linkage group AC04, and has been identified as possessing a fusion polymorphism in Arctic charr (Moghadam *et al.* 2007). Our data also suggest that a two acrocentric *vs.* one metacentric polymorphism exists for AC04 (see below). The q-arm in this chromosome set involves a fusion of two ancestral salmonid chromosome arms that are homologous to Omy02q and Omy25, while the p-arm is homologous to Omy24. None of these arms are homeologous to one another, and are therefore unlikely to show pseudolinkage affinities. The second set of chromosomes (AC20 group) involves a small (AC20a) and a large (AC20b) acrocentric arm, where the large arm appears to be composed of two fused ancestral salmonid arms that are homologous to Omy12p/q and Ots09/q. Therefore, it is more likely that AC20b represents an entire metacentric chromosome, and AC20a represents a separate acrocentric arm, rather than AC20a/20b representing a large metacentric chromosome comprised of joined homeologous arms (Woram *et al.* 2004). This would support the suggestion that there are 10 stable metacentrics in North American Arctic charr (Hartley 1989; Phillips *et al.* 2002), with the AC04 polymorphism generating an additional metacentric in some individuals. The homologous chromosome arm of AC20a in rainbow trout is Omy13q, and, since Omy12q/Omy13q are homeologs, they may show pseudolinkage affinities. Twenty-seven linkage groups appear to be acrocentric, including two (AC04 and AC17) that result from a tandem fusion of qa and qb chromosome arms. AC17 appears to include segments homologous to both Omy16q and 20q. The total chromosome arm number (NF) observed was 100, containing 52 haploid ancestral arm segments.

**Table 2:** Salmonid chromosome arm homologies referenced to Arctic charr linkage group homologies

Ssa Homeolog Pair*^a^*	Atlantic Salmon	Rainbow Trout*^b^*	Arctic Charr	Chinook Salmon*^c^*	Ssa Homeolog Pair	Atlantic Salmon	Rainbow Trout	Arctic Charr	Chinook Salmon
10qa/b	Ssa16qa	Omy01p^15^	AC26	Ots06p	27	Ssa14qb	Omy14p	AC30	Ots31
01qa/b	Ssa18qa^14^	Omy01q^14^	AC25	Ots06q^14^	09qb	Ssa05p	Omy14q	AC06q	Ots21
02p^1^	Ssa05q^1^	Omy02p^1^	AC06p	Ots23^1^	19qa	Ssa29^8^	Omy15p	AC27q	Ots29^8^
16qa&23	Ssa10qb	Omy02q^15^	AC04qb	Ots19	07q^7^	Ssa17qb^7^	Omy15q^7^	AC24	Ots17^7^
05q^1^	Ssa02p^1^	Omy03p^1^	AC35	Ots03p^1^	28&29	SSa19qb^8^	Omy16p^16^	AC18p	Ots24^8^
21	Ssa25	Omy03q^9^	AC02	Ots03q	15qb	Ssa13qa^1^	Omy16q^1^	AC17qa	Ots22^1^
10qa/b	Ssa23	Omy04p^10^	AC13p	Ots01p	22	Ssa12qb	Omy17p^11^	AC01p	Ots02p
15qa	Ssa06q	Omy04q	AC14q	Ots18	12qa^6^	Ssa02q^6^	Omy17q^6^	AC01q	Ots02q^6^
11qb&13qb	Ssa01qb	Omy05p	AC29	Ots20	17qa^3^	Ssa16qb^3^	Omy18p^3^	AC27p	Ots14p^3^
23	Ssa10qa	Omy05q^10^	AC16	Ots05q	14qb	Ssa27	Omy18q	AC31	Ots13p
20qa	Ssa24	Omy06p	AC15p	Ots04p	08q/04p^4^	Ssa04p/08q^4^	Omy19p^4^	AC34	Ots11p^4^
11qa^2^	Ssa26^2^	Omy06q^2^	AC15q	Ots04q^2^	09qa	Ssa01p	Omy19q	AC09	Ots11q
16qb^3^	Ssa17qa^3^	Omy07p^3^	AC12	Ots07p^3^	05p/19qa/b&01qa	Ssa09qb/28	Omy20p^16^	AC08q	Ots25
12qb	Ssa22	Omy07q^11^	AC11	Ots07q	29	Ssa19qa	Omy20q	AC17qb	Ots25
06q	Ssa15qa	Omy08p	AC28	Ots05p	18qb	Ssa07p^7^	Omy21p^7^	AC03p	Ots15p^7^
03p	Ssa14qa	Omy08q^12^	AC32	Ots10q	17qb^7^	Ssa07q	Omy21q^13^	AC03q	Ots15q
07p	Ssa18qb	Omy09p^13^	AC37	Ots10p	25	Ssa21	Omy22p^9^	AC36	Ots26
13qa	Ssa15qb^1^	Omy09q^1^	AC07	Ots16q^1^			Omy22q		
13qb	Ssa04q	Omy10p	AC23	Ots30	28&29	Ssa01qa^14^	Omy23^14^	AC18q	Ots01q^14^
04p/08q^4^	Ssa08q/04p^4^	Omy10q^4^	AC13q	Ots34^4^	20qb	Ssa09qc	Omy24	AC04p*^d^*	Ots14q
24/29	20qa/19qa	Omy11p	AC14p	Ots16p/12p	01p	Ssa09qa	Omy25	AC04qa	Ots08p
24	Ssa20qa	Omy11q	AC33	Ots12p	26^2^	Ssa11qa^2^	Omy26^2^	AC10	Ots12q^2^
04q	Ssa13qb	Omy12p	AC20b-2	Ots09p	09qc	Ssa20qb	Omy27	AC22	Ots13q
06q/01qb&04q^5^	03q/13qb^5^	Omy12q^5^	AC20b-1	Ots09q^5^	14qa	Ssa03p	Omy28^12^	AC19	Ots28
02q^6^	Ssa12qa^6^	Omy13p^6^	AC21	Ots32^6^	05p/19qa/b&01qa	Ssa09qb/28	Omy29	Ac08p	Ots08q
03q^5^	Ssa06p^5^	Omy13q^5^	AC20a	Ots27^5^	01qb&4p	Ssa11qb	Sex	AC05	Ots33

These were determined based on the most common BLASTn hit locations of a linkage group’s markers when compared to the Atlantic salmon genome and the rainbow trout draft genome. Additionally, homologies with Chinook salmon chromosome arms are presented based on known homologies in Atlantic salmon and rainbow trout, though direct BLASTn comparison of the Arctic charr linkage map and Chinook salmon genome was not performed. The column “Ssa Homeolog pair” shows each chromosome arm’s homeolog partner derived from a common pre-Ss4R ancestor.

a Cells in columns 1 and 6 with matching superscript numbers represent HRTA identified in Atlantic Salmon [data from Lien *et al.* (2016)].

b Homeolog pairs identified in rainbow trout based on high numbers of duplicate markers have matching superscript numbers in columns 3 and 8 [data from Danzmann *et al.* (2008) and Berthelot *et al.* (2014)].

c Homeolog pairs identified in Chinook salmon based on high numbers of duplicate markers have matching superscript numbers in columns 5 and 10 [data from Brieuc *et al.* (2014)].

d Indicates sex the linkage group of Arctic charr.

### Sex-linkage group polymorphism in AC04

Previous work has shown AC04 to be polymorphic, and taking the form of either a single linkage group (type 1) or two unlinked linkage groups (type 2) (Moghadam *et al.* 2007). The mapping parents in this study are both type 2 individuals; microsatellite markers associated with AC04 are found on both AC04p and AC04q (Woram *et al.* 2004; Moghadam *et al.* 2007; Timusk *et al.* 2011). Previous research identified three salmonid chromosome arms homologous with AC04 (Timusk *et al.* 2011). Two of these arms are homologous with AC04q, and the third is homologous with AC04p (Table 2). AC04p also contains the sex-determining gene, sdY (Yano *et al.* 2013). Although two separate, type 2 AC04 linkage groups are observed in the current linkage map, the polymorphic nature of this linkage group, and the evidence presented here, is indicative of a fusion/fission polymorphism for the sex-determining chromosome of Arctic charr. Affinity of the sdY marker to the Slml-family of TEs supports the suggestion that the reported sex-linkage difference between North American (AC04) and European (AC01/21) Arctic charr may be the result of a translocation through TE movements (Woram *et al.* 2003; Küttner *et al.* 2011).

### Pseudolinkage

Two pairs of homeologous linkage groups (AC01q/21 and AC13q/34) were detected as possessing a weak pseudolinkage to one another (LOD ≥ 3–5). Interestingly, pseudolinkage was detected between both homeologous pairs of linkage groups in both the male and female parents (Table 3). Previously, pseudolinkage was thought to occur only within male meiosis, with rare reports of female pseudolinkage (Ostberg *et al.* 2013; Allendorf *et al.* 2015). Our identification of pseudolinkage in a female confirms that multivalents are also likely formed during female meioses.

**Table 3:** Allele counts of the two homeolog pairs displaying pseudolinkage within females

Marker Pair		Genotypes		Linkage Group		Parental Phases (Marker A/Marker B)		Recombinant Phases (Marker A/Marker B)		Chi-Squared	*P*-value
Marker A	Marker B	Marker A	Marker B	Marker A	Marker B	Alleles	Count	Alleles	Count		
TP47181	TP21253	G,C	A,G	AC13	Pseudolink	G/A	32	C/A	23	18.7	3.20E−04
						C/G	25	G/G	5		
TP21253	TP30908	A,G	T,C	Pseudolink	AC34	A/T	43	A/C	12	36	7.50E−08
						G/C	23	G/T	7		
TP47181	TP30908	G,C	T,C	AC13	AC34	G/T	23	C/T	27	4.2	0.243
						C/C	21	G/C	14		
TP10591	TP15996	T,G	G,A	AC21	Pseudolink	T/G	14	G/G	4	15.8	0.0013
						G/A	13	T/A	1		
TP15996	TP32826	G,A	A,T	Pseudolink	AC01	G/A	18	A/A	0	33	3.20E−07
						A/T	14	G/T	0		
TP10591	TP32826	T,G	A,T	AC21	AC01	T/A	22	G/A	23	7.9	0.049
						G/T	27	T/T	10		

These instances appear to result from an excess of parental phase genotypes. Flanking markers from both linkage groups with the most complete genotypes, along with the principle marker causing pseudolinkage, are displayed. Note, for AC21/AC01, the marker causing pseudolinkage (TP15996) was heterozygous in both parents (ab X ab cross). Therefore, the phases for half of the progeny could not be ascertained. Chi-squared goodness of fit tests were performed for each pair of alleles, comparing the observed genotype frequencies to a null hypothesis of a 1:1:1:1 genotype distribution.

Pseudolinkage is a phenomenon arising due to segregation of gametes following preferential pairing of homeologous chromosome arms during meiosis I (Allendorf *et al.* 2015; May and Delany 2015). Preferential as opposed to random pairing typically occurs in cases of hybridization, where the homeolog pairs provided by one parent may be more closely related to one another due to their species-specific ancestry. Therefore, within the hybrid, the homeologs from one parent may therefore be more likely to pair with one another and recombine. Crossing-over tends not to occur between homeologous markers located very close to the centromere. If preferential pairing occurs at meiosis I, this leads to a significant excess of nonparental genotypes being produced following meiosis II, because alternate disjunction occurs as the multivalents separate at meiosis I [see Allendorf *et al.* (2015) and May and Delany (2015) for a more detailed explanation of these models]. This produces a statistical linkage between the two homeologous chromosome arms characterized by a significant excess of nonparental gametes, and is a characteristic feature of hybrid salmonids.

Random pairing of homeologous pairs may, however, still occur as the genome undergoes the process of diploidization within species. This may involve the random formation of both bivalents, and multivalents, between the homeologous pairs, such that gametic expectation models cannot be precisely defined. This may result in varying levels of exchange among alleles for loci that are located proximal and distal to chiasmata junctions along the length of randomly paired homeologous chromosomes [see Sakamoto *et al.* (2000), for an explanation]. We have developed models to explain this weaker form of pseudolinkage, and will present these in a subsequent publication.

### Homeologies

**Figure 3:**
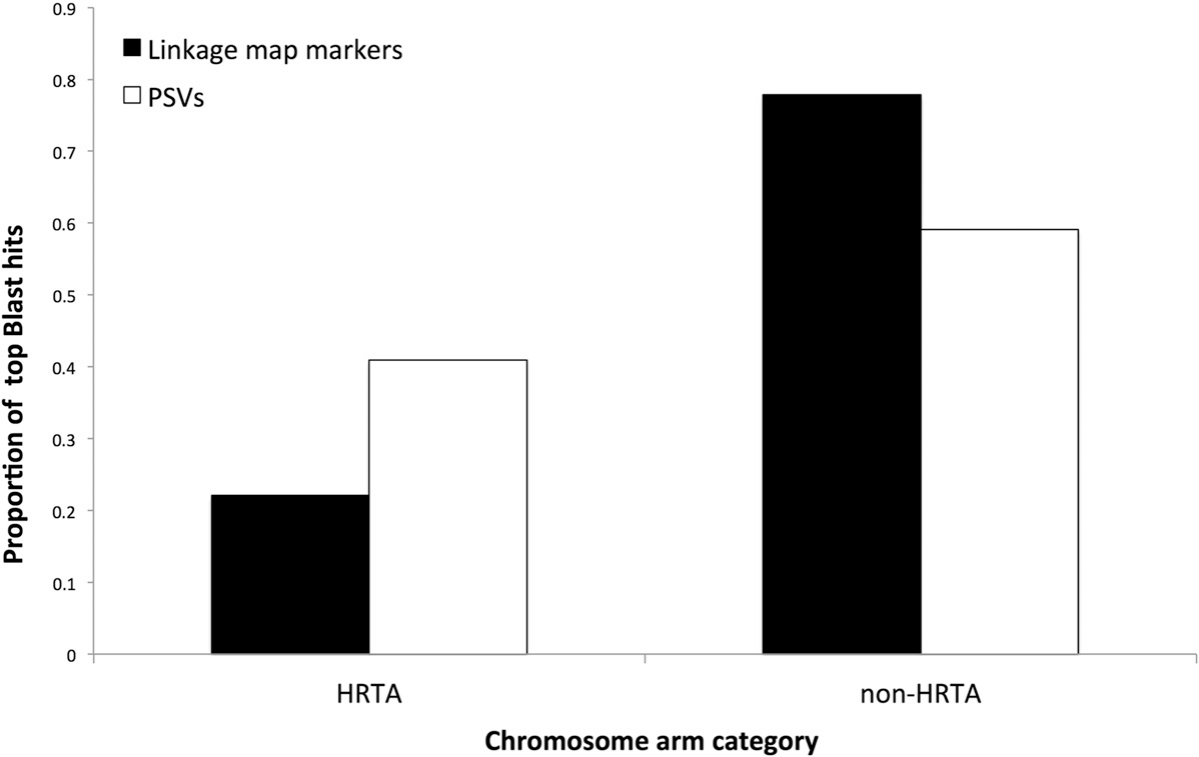
BLASTn hit locations in the Atlantic salmon genome for Arctic charr PSVs, and linkage map markers; 40.8% (462/1130) of PSVs had their top hit locations on HRTAs, while only 22.1% (950/4290) of the linkage map SNPs had their top Blast hit locations on HRTAs.

Arm homologies identified in Atlantic salmon, rainbow trout, and chinook salmon (Table 2) with Arctic charr, were used to identify homeologous arm pairings within Arctic charr (Table 4). Lower levels of duplicate marker regions were detected for certain arms, but can nonetheless be inferred based upon the recent extensive survey of duplicate gene copies in Atlantic salmon (Hermansen *et al.* 2016) (Table 4). All seven homeologous pairs in Arctic charr identified as HRTAs in other species contain one chromosome arm that has undergone a chromosome fusion. One pair of HRTA identified in Atlantic salmon (Ssa09qc/20qb) (Lien *et al.* 2016) did not show a high number of duplicates in either rainbow trout or chinook salmon. Similarly a HRTA region in both rainbow trout and chinook salmon (Omy01q and Ots06q/Omy23 and Ots01q), was not identified as an HRTA in Atlantic salmon (Table 2). Four AHPs, where neither homeolog has undergone a fusion event since the Ss4R, were identified in Arctic charr (Table 5). AHP likely undergo minimal residual tetrasomic inheritance, given that AHP multivalent pairings are unlikely due to structural instability (May and Delany 2015). We found support for this prediction in that the number of PSVs aligning to AHPs was significantly lower compared to the number aligning to the other chromosome arms (*P* < 0.0001). Of the linkage map markers that successfully aligned to the Atlantic salmon genome, 20% (861/4290) aligned to HRTA chromosome arms, while only 14.0% of PSVs (158/1130) had top hit locations on AHPs. These results suggest that fewer duplicate loci exist on AHPs, which might be indicative of more rapid diploidization relative to the rest of the genome (Figure 3).

**Table 4:** Reference table of homeologous chromosome pairs in Arctic charr

Homeolog 1	Homeolog 2
AC01p	AC11
AC01q	AC21
AC02	AC36
AC03p	AC24
AC03q	AC37
AC04p	AC14p/AC33*^a^*
AC04p	AC22
AC04qa	AC09
AC04qb	AC26
AC06	AC08p/AC08q
AC06p	AC35
AC07	AC17qa
AC08p	AC18p?*^a^*
AC10	AC15q
AC12	AC27p
AC13p	AC16
AC13q	AC34
AC14q	AC28*^a^*
AC15p	AC14p/AC14q/AC33*^a^*
AC17qb	AC27q
AC18p	AC27q
AC18q	AC25
AC19	AC32
AC20b-1	AC20a
AC23	AC04qb/AC05/AC20b-1/AC-20b-2*^a^*
AC29	AC05/AC20b-2*^a^*
AC30	AC31

a Identified through their homologies in Atlantic salmon, and homeologies identified in Hermansen *et al.* (2016).

**Table 5:** Reference table for the analysis of Arctic charr duplicate loci distribution

Atlantic Salmon Chromosome Arm	Arctic Charr Linkage Group	High Residual Tetrasomy Arms (HRTA)	Acrocentric Homeolog Pairs (AHP)	Atlantic Salmon Chromosome Arm	Arctic Charr Linkage Group	High Residual Tetrasomy Arms (HRTA)		Acrocentric Homeolog Pairs (AHP)
Ssa01p	AC09			Ssa13qa	AC17qa			
Ssa01qa	AC18q			Ssa13qb	AC20b-2			
Ssa01qb	AC29		AHP	Ssa14qa	AC32		AHP	
Ssa02p	AC35	HRTA		Ssa14qb	AC30		AHP	
Ssa02q	AC01q	HRTA		Ssa15qa	AC28			
Ssa03p	AC19		AHP	Ssa15qb	AC07			
Ssa03q	AC20b-1/AC20b-2	HRTA		Ssa16qa	AC26			
Ssa04p	AC13q/AC-34	HRTA		Ssa16qb	AC27p	HRTA		
Ssa04q	AC23			Ssa17qa	AC12	HRTA		
Ssa05p	AC06q			Ssa17qb	AC24	HRTA		
Ssa05q	AC06p	HRTA		Ssa18qa	AC25			
Ssa06p	AC20a	HRTA		Ssa18qb	AC37			
Ssa06q	AC14q			Ssa19qa	AC17qb/AC14p			
Ssa07p	AC03p			Ssa19qb	AC18p			
Ssa07q	AC03q	HRTA		Ssa20qa	AC14p/AC33			
Ssa08q	AC13q/AC34	HRTA		Ssa20qb	AC22			
Ssa09qa	AC04qa			Ssa21	AC36		AHP	
Ssa09qb	AC08p/AC08q			Ssa22	AC11			
Ssa09qc	AC04p			Ssa23	AC13p			
Ssa10qa	AC16			Ssa24	AC15p			
Ssa10qb	AC04qb			Ssa25	AC02		AHP	
Ssa11qa	AC10	HRTA		Ssa26	AC15q	HRTA		
Ssa11qb	AC05		AHP	Ssa27	AC31		AHP	
Ssa12qa	AC21	HRTA		Ssa28	AC08p/AC08q			
Ssa12qb	AC01p			Ssa29	AC27q			

The Atlantic salmon chromosome arms are listed alongside their Arctic charr homologs. The table lists all chromosome arms classified as belonging to the HRTA or AHP categories.

### Duplicate loci

The distribution of PSVs throughout the Arctic charr genome appeared to be nonrandom, with certain regions preserving higher numbers of duplicate loci. BLASTn alignment of PSVs, and linkage map SNPs to the Atlantic salmon genome, was performed to compare the locations of linkage map markers and PSVs. The top BLASTn hit locations (based on lowest *e*-values) were used to assess the distribution of SNPs throughout the genome. Direct comparison of the markers through linkage mapping was not possible, given that PSVs lack any type of segregation pattern, and therefore cannot be mapped. For markers with BLASTn hits of equal *e*-values on a single chromosome arm, only a single hit was used per chromosome arm in the dataset. In the case of markers (both linkage map markers and PSVs) with BLASTn hit locations of equal *e*-values on two Atlantic salmon chromosomes, a BLASTn hit to each chromosome arm was retained in the dataset, as these markers represented potential duplicate loci. Markers with equal BLASTn hits to three or more locations were excluded from the dataset.

**Figure 4:**
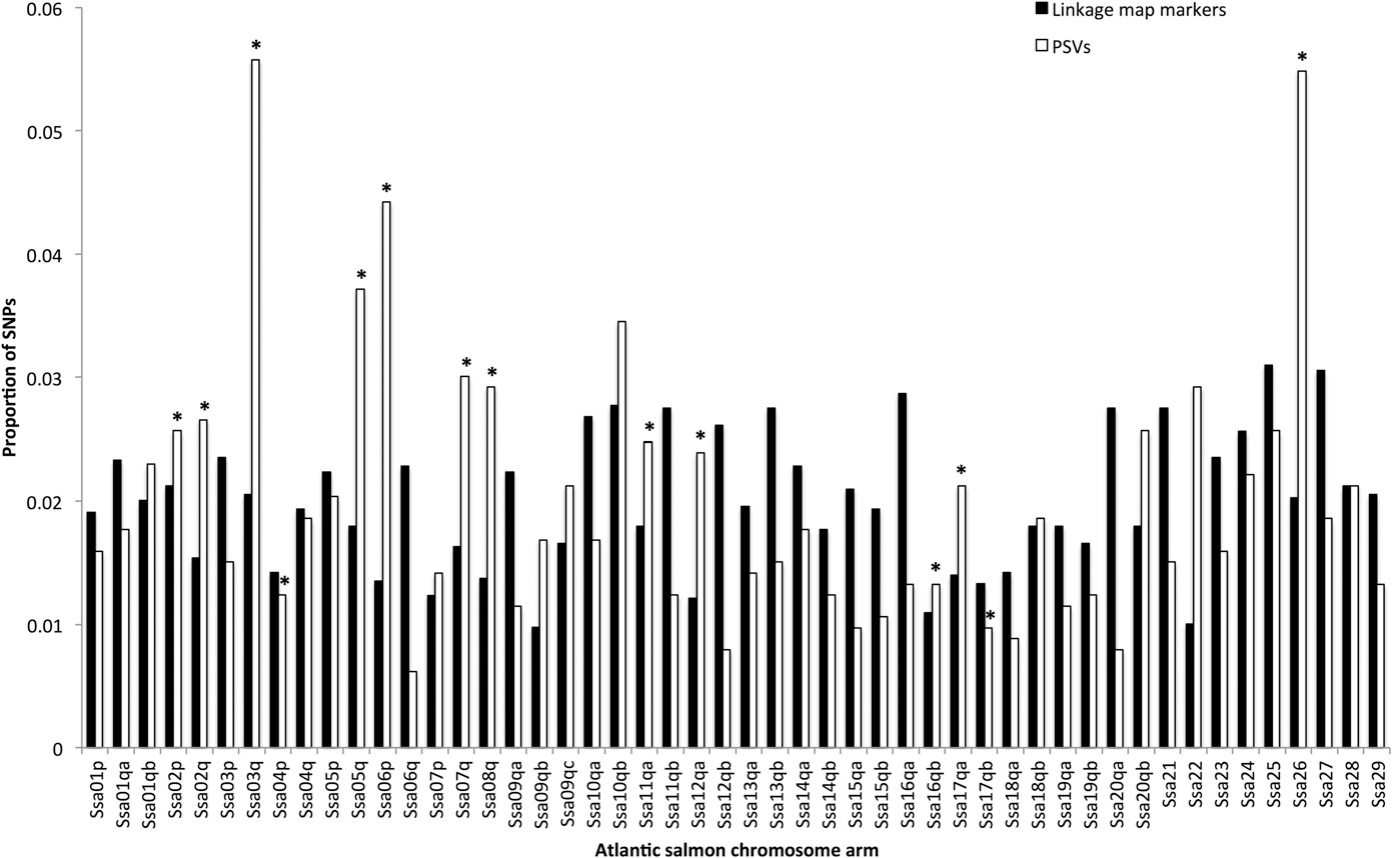
The top BLASTn hit locations of Arctic charr linkage map SNPs, and Arctic charr PSVs, across Atlantic salmon chromosome arms. The data are shown in the proportion of hits from a category (Map SNPs or PSVs) in order to account for the bias of Atlantic salmon chromosome size. Arms with a * are HRTAs identified in Lien *et al.* (2016).

The analysis identified 1130 PSV Blast hits in the Atlantic salmon genome (Figure 4), in addition to the 4429 SNPs assigned to the linkage map. If the PSV distribution throughout the genome is random, and chromosome arm size was the only factor influencing their distribution, then the proportion of PSVs aligning to each chromosome would be similar to the proportion of linkage map SNPs aligning to each chromosome arm. To assess the effect of genomic architecture on the preservation of duplicate loci, chromosome arms were binned into two categories: HRTAs (listed in Table 5) and non-HRTA (other) salmonid chromosome arms. A contingency test showed higher numbers of PSVs aligned to the HRTAs than expected (*P* < 0.0001) (Figure 3). Of the 14 chromosome arms in the HRTA category, 12 had higher proportions of PSV Blast hits than linkage map marker Blast hits (Figure 4). The two HRTA arms with lower numbers of PSV BLASTn hits (Ssa04p and Ssa17qb) had homeologs with high numbers of PSV hits (Ssa08q and Ssa07q, respectively). This suggests that the lower number of PSV hits on the HRTA arms may be due to more PSVs aligning to their homeologs because of small sequence differences. High numbers of PSVs are observed in regions that preserved residual tetrasomy longer after the Ss4R than other parts of the genome, which may be indicative of historically reduced diploidization rates (Brieuc *et al.* 2014).

Several other Atlantic salmon arms displayed high numbers of PSV BLASTn hits, notably Ssa09qb, Ssa09qc, Ssa10qb, and Ssa22. The Arctic charr homologs of these arms are AC08p/q, AC04p, AC04qb, and AC11, respectively. All of these arms, or their homeologs in Arctic charr, have undergone a fusion. Interestingly, these arms also share homology to rainbow trout chromosome arms with high numbers of duplicates (Table 2). Previously, it had been thought that one metacentric chromosome must be present in a homeolog pair to provide the stability necessary for homeologous pairing, and multivalent formation (Kodama *et al.* 2014). However, Lien *et al.* (2016) have recently shown that metacentric structures are not a requirement for homeologous pairing, given that two HRTA pairs in Atlantic salmon (Ssa11qa/Ssa26 and Ssa16qb/Ssa17qa) preserve high sequence similarity without the presence of a metacentric, but, in both cases, one of the arms has undergone a fusion event. This suggests that fused acrocentrics, as well as metacentric chromosomes, provide the structural stability necessary for homeologous recombination.

The conservation of duplicate loci on HRTAs does not appear to be due to current chromosome structure, because the HRTAs do not display homologous chromosome arm fusions across species (with the exception of AC03/Ssa07/Omy21/Ots15). Therefore, the slower diploidization in these regions may be attributed to some aspect of their evolutionary past. For instance, certain fusions may have arisen in the common ancestor of all these salmonid lineages following the Ss4R. This could have provided the HRTA homeolog pairs with the ability to form multivalents during meiosis, and undergo residual tetrasomy, thereby slowing diploidization rates. A large number of species-specific fusion/fission events since the more recent divergence of salmonids (Macqueen and Johnston 2014) could disjoin previous chromosome structures, and also explain why the seven HRTA homeolog pairs preserve duplicate loci in multiple species, despite the large variation in karyotypic structures across current salmonid species.

To search for possible duplicate SNP positions that could have arisen from the Ss4R event, we reciprocally BLASTn aligned all linkage map SNP markers against each other, and retained those duplicate pairs that shared ≥95% identity to one another, as well as retaining ≥95% of an overlap in their length distributions. Surprisingly, of the 362 duplicate pairs identified, only 18 pairs were interchromosomal duplicate pairs. Of these 18 pairs, only three were considered to be possible WGD paralogs, as the other 15 pairs involved either one or both SNPs with TE-specific sequence (see File S4). One of the potential paralogs that was not associated with TE, involved markers from identified homeolog pair of AC03q/37. Two of the potential paralogs not associated with TEs involved markers from the linkage groups AC06p and AC32, which is not an identified homeolog region in the Arctic charr map. However, these two paralog sets show homology to the Ssa02p/05q paralogs, and therefore may indicate a small region of previously unidentified homeology.

The vast majority of duplicate pair markers appear to be intrachromosomal duplicates (95.1%) (File S4). To assess whether chromosome structure may have had an influence on the distribution of these duplicates, we compared the proportion of duplicated SNP markers between metacentric *vs.* acrocentric type linkage groups. Duplicates within AC04p and AC04q were included in the metacentric grouping, as well as duplicates within AC17, given that this acrocentric appears to be composed of fused chromosome arms. No differences were detected in the distribution of duplicates between the chromosome types (*P* > 0.05) (Table 6).

**Table 6:** Arctic charr linkage map location of duplicate pair SNPs that shared ≥95% identity with one another

Linkage Group (LG)	Inter-LG Pair Members*^a^*	Intra-LG Duplicates	Proportion Marker Duplicates*^b^*	Linkage Group (LG)	Inter-LG Pair Members	Intra-LG Duplicates	Proportion Marker Duplicates
AC01	4	30	0.192	AC20a	2	12	0.15
AC02	0	20	0.149	AC20b	0	28	0.136
AC03	3	16	0.149	AC21	0	18	0.125
AC04p	1	22	0.224	AC22	0	10	0.125
AC04q	3	30	0.133	AC23	1	22	0.206
AC05	0	16	0.136	AC24	0	8	0.114
AC06	4	26	0.129	AC25	2	16	0.219
AC07	0	8	0.108	AC26	3	32	0.219
AC08	1	12	0.115	AC27	0	24	0.192
AC09	0	12	0.136	AC28	0	6	0.086
AC10	0	6	0.08	AC29	1	16	0.2
AC11	0	0	0	AC30	1	8	0.143
AC12	0	2	0.044	AC31	0	22	0.204
AC13	0	30	0.211	AC32	3	16	0.121
AC14	0	20	0.099	AC33	1	30	0.2
AC15	0	20	0.12	AC34	0	2	0.043
AC16	3	26	0.183	AC35	0	22	0.256
AC17	0	16	0.16	AC36	0	8	0.075
AC18	0	28	0.175	AC37	3	30	0.133
AC19	0	24	0.233				

a Number of SNPs where the duplicate member maps to a different linkage group.

b Number of duplicates/total number of SNPs mapped to the LG.

Duplicate marker pairs do not map next to one another within linkage groups, suggesting that they are not tandem repeats. These marker duplicates may be part of larger segmental duplicate blocks that can vary in size from 1 to 400 kb (Mendivil Ramos and Ferrier 2012), but alignment of these regions to more complete scaffold contigs would be needed to test this idea. The reason so few apparent WGD paralog regions were detected is likely due to the high stringency in which duplicated regions were identified. Interchromosomal 4R paralogs have average identity levels ranging from 86 to 90% (Moghadam *et al.* 2011; Berthelot *et al.* 2014), with average levels up to 96% for protein-coding duplicate regions (Berthelot *et al.* 2014). Hence, an analysis using lowered stringency cut-offs will likely reveal higher frequencies of WGD paralog regions than those reported here.

Many of the duplicate markers appear to align to identical locations within both the rainbow trout and Atlantic salmon genomes, but in opposite strand orientations (see File S4). Upon closer inspection of these duplicates, we observed that several reads aligning to each contig cluster were longer in either the 5′ or 3′ direction, and these reads spanned an *Eco*T22I cut-site on either side. Both the 3′ side of the forward strand, and 5′ side of the reverse strand were bordered by the 5 bp signature of a DNA strand cut by EcoT22I. This suggests that an internal cut-site may have been skipped due to an epigenetic modification, and that an additional EcoT22I cut-site, 20–30 bp away, flanked the uncut site.

We queried all 425,391,431 reads obtained from the fish used in this study, and determined that the presence of uncut EcoT22I sites averaged 2.8%, while those found in the paired duplicates having extended reads ranged from 4 to 6% supporting the suggestion that these regions may be more epigenetically modified. However, many of these reads also appeared as chimeric religations, highlighting that enzymes susceptible to epigenetic modification may lead to genotyping errors (Jiang *et al.* 2016).

### Distribution of Arctic charr duplicated markers in relation to the Atlantic salmon genome

**Figure 5:**
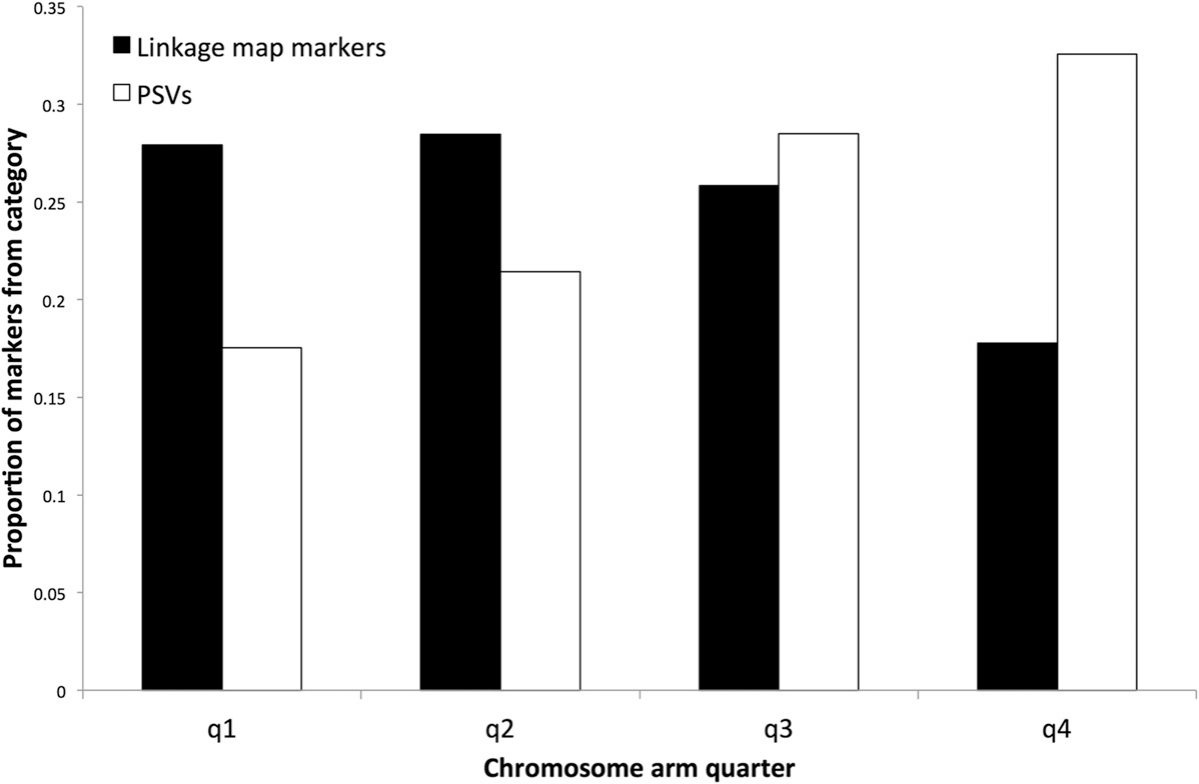
Distribution of the top Blast hits of Arctic charr linkage map SNPs and PSVs across Atlantic salmon chromosome arms. q1 is the quarter closest to the centromere, q4 is the quarter closest to the telomere.

BLASTn alignments of the Arctic charr duplicated markers were not uniformly distributed along the length of Atlantic salmon chromosomes from the Lien *et al.* (2016) assembly. Significantly more Arctic charr PSVs aligned to the 4th quarter (telomeric ends) of chromosome arms (*P* < 0.0001) (Figure 5). This suggests that PSVs are preserved near the telomeres in the regions of chromosomes that undergo residual tetrasomy (Wright *et al.* 1983; Allendorf *et al.* 2015; May and Delany 2015). Previous studies in salmonids have also observed that duplicate loci are present in higher frequencies near the telomeres (Sakamoto *et al.* 2000; Brieuc *et al.* 2014; Kodama *et al.* 2014; Larson *et al.* 2015; McKinney *et al.* 2015; Waples *et al.* 2015). There was also a significant reduction of linkage map markers with top Blast hits in the 4th quarter of chromosomes, which suggests that residual tetrasomy may generate high numbers of duplicate loci that cause telomeric regions to be under-represented with markers (Allendorf *et al.* 2015).

### Transposon activity

**Figure 6:**
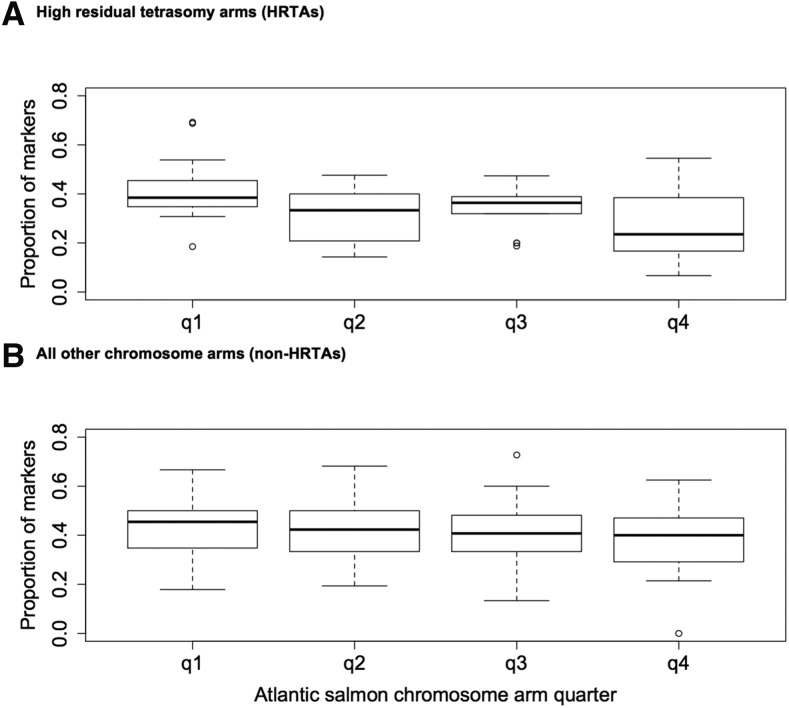
Proportion of Arctic charr Map SNPs and PSVs with TE hits across the length of chromosome arms. SNPs were grouped based on their alignment to Atlantic salmon chromosome arms. q1 is the quarter closest to the centromere, q4 is the quarter closest to the telomere. Atlantic salmon chromosome arms were grouped according to whether they were HRTAs (A) or non-HRTAs (B).

Recent studies of salmonid genomes have observed lower instances of sequence homology to TEs within genomic regions characterized as preserving tetrasomy (McKinney *et al.* 2015; Waples *et al.* 2015; Lien *et al.* 2016). We assessed TE distributions through BLASTn alignments to the Atlantic salmon genome to determine if chromosomes homologous to HRTA had lower levels of TE alignments. Of the 4405 linkage map SNPs, 1608 (36.5%) had homology with transposable elements in Repbase Update’s list of known vertebrate TEs (Jurka *et al.* 2005). This was a lower proportion of TE hits than expected as repetitive elements comprise 58–60% of the Atlantic salmon genome, and suggests that our dataset underrepresents the proportion of repetitive elements in Arctic charr. We detected a small but significant reduction in TE activity between HRTAs (30.9% of SNPs on these arms had significant TE hits), and non-HRTA chromosome arms (35.8%) (*P* = 0.0009) (Table 7). Using the Atlantic salmon genome as a scaffold, we detected no significant difference in the frequency of TE hits between the telomeric and centromeric regions of chromosomes (*P* = 0.1322) (Figure 6).

**Table 7:** TE distribution of the SNPs aligning to the Atlantic salmon chromosome arms, and PSVs markers not found in the linkage map

	No TE Hits	TE Hits
Linkage groups containing HRTAs	976	436
Linkage Groups with no HRTAs	2572	1436
Total	3548	1872

There was a slight but significant reduction in TE activity seen on HRTA (30.9% of SNPs with significant TE hits) relative to all other chromosome arms (35.8% with significant TE hits) (*P* = 0.0009).

### Conclusion

We have presented a SNP-based linkage map of the Arctic charr genome, which is comprised of 4508 markers spanning 39 linkage groups. The map was used to identify the chromosome type of each linkage group, and the homologous chromosome arms in other salmonid species. Using data from the Atlantic salmon genome, we have identified putative homeologous arm pairs in Arctic charr. Based on the distribution of PSV, we suggest that genomic architecture is influencing diploidization rate in the Arctic charr genome, with higher levels of duplicate loci being preserved on HRTAs and lower numbers of duplicate loci preserved on AHPs. Transposon activity was also quantified, but we failed to detect a strong influence of genomic architecture on TE distribution. Pseudolinkage was also detected in both the male and female parents, and this involved two HRTA homeolog pairs (AC01q/21 and AC13q/34). This map also characterized the genome of a salmonid species with a more basal karyotype, and shows how these differences in genomic architecture have influenced diploidization.

## Supplementary Material

Supplemental material is available online at www.g3journal.org/lookup/suppl/doi:10.1534/g3.116.038026/-/DC1.
