# Supplementary material for: A SNP Based Linkage Map of the Arctic Charr (Salvelinus alpinus) Genome Provides Insights into the Diploidization Process After Whole Genome Duplication
Source: G3 (Bethesda). 2016 Dec 16;7(2):543–56. doi: 10.1534/g3.116.038026 (PMC5295600; doi:10.1534/g3.116.038026)
Supplement: Supplementary file 1 [file 543FileS1.docx]

File S1. List of all Arctic charr SNPs and associated sequence data. (.xlsx, 309 KB)

<http://www.g3journal.org/lookup/suppl/doi:10.1534/g3.116.038026/-/DC1/FileS1.xlsx>
