# Supplementary material for: A SNP Based Linkage Map of the Arctic Charr (Salvelinus alpinus) Genome Provides Insights into the Diploidization Process After Whole Genome Duplication
Source: G3 (Bethesda). 2016 Dec 16;7(2):543–56. doi: 10.1534/g3.116.038026 (PMC5295600; doi:10.1534/g3.116.038026)
Supplement: Supplementary file 5 [file 543FileS3.docx]

File S3. Top BLASTn hit locations for Arctic charr map SNPs and PSVs when aligned to the Atlantic salmon genome, rainbow trout genome and Repbase Update’s list of vertebrate transposable elements. (.xlsx, 1.3 MB)

[http://www.g3journal.org/lookup/suppl/doi:10.1534/g3.116.038026/-/DC1/FileS3.xlsx](http://www.g3journal.org/lookup/suppl/doi:10.1534/g3.116.038026/-/DC1/FileS2.xlsx)
