# Supplementary material for: A SNP Based Linkage Map of the Arctic Charr (Salvelinus alpinus) Genome Provides Insights into the Diploidization Process After Whole Genome Duplication
Source: G3 (Bethesda). 2016 Dec 16;7(2):543–56. doi: 10.1534/g3.116.038026 (PMC5295600; doi:10.1534/g3.116.038026)
Supplement: Supplementary file 7 [file 543FileS4.docx]

File S4. Blastn data for duplicate marker pairs. (.xlsx, 124 KB)

<http://www.g3journal.org/lookup/suppl/doi:10.1534/g3.116.038026/-/DC1/FileS4.xlsx>
